# Preparation of Monoclonal Antibody for Brevetoxin 1 and Development of Ic-ELISA and Colloidal Gold Strip to Detect Brevetoxin 1

**DOI:** 10.3390/toxins10020075

**Published:** 2018-02-08

**Authors:** Sumei Ling, Shiwei Xiao, Chengjie Xie, Rongzhi Wang, Linmao Zeng, Ke Wang, Danping Zhang, Xiulan Li, Shihua Wang

**Affiliations:** Key Laboratory of Pathogenic Fungi and Mycotoxins of Fujian Province, Key Laboratory of Biopesticide and Chemical Biology of Education Ministry, and School of Life Sciences, Fujian Agriculture and Forestry University, Fuzhou 350002, China; wshyyl@sina.com (S.L.); xiaoshiwei34@163.com (S.X.); xiec7@foxmail.com (C.X.); WRZ0629@126.com (R.W.); 18750114951@163.com (L.Z.); rebekah0926@hotmail.com (K.W.); zhang1119383881@sina.com (D.Z.); 18850136475@163.com (X.L.)

**Keywords:** Brevetoxin-1, monoclonal antibody, ELISA, colloidal gold strip

## Abstract

Brevetoxin-1 (BTX-1), a marine toxin mostly produced by the dinoflagellatae *Karenia brevis*, has caused the death of marine organisms and has had numerous toxicological effects on human health. Hence, it is very necessary to develop a rapid, economical, and reliable immunoassay method for BTX-1 detection. In this study, two kinds of complete antigen were synthesized using the succinic anhydride and isobutyl chloroformate two-step methods. Conjugate BTX-1-OVA was used as an antigen for mice immunization, and BTX-1-BSA for measuring the titer of the produced antibodies. A hybridoma cell line 6C6 stably secreting monoclonal antibody (mAb) against BTX-1 was obtained by fusing SP2/0 myeloma cells with the spleen cells from the immunized mouse. The hybridoma 6C6 was injected into the abdomen of BALB/c mice to obtain ascites, and the anti-BTX-1 mAb was harvested from ascites by precipitation with caprylic acid/ammonium sulfate (CA-AS). The anti-BTX-1 mAb was identified as an IgG1 subtype, and the cross-reactivity results showed that anti-BTX-1 mAb was highly specific to BTX-1 with the affinity of 1.06 × 10^8^ L/mol. The indirect competitive ELISA results indicated that the linear range for BTX-1 detection was 14–263 ng/mL with IC_50_ of 60 ng/mL, and a detection limit of 14 ng/mL. The average recovery rate from the spiked samples was 88 ± 2% in intra-assay and 89 ± 2% in inter-assay. The limit of detection (LOD) using the colloidal gold strip was 200 ng/mL with high specificity. Therefore, the anti-BTX-1 mAb can be used to detect BTX-1 in shellfish and other related samples.

## 1. Introduction

The toxic photosynthetic dinoflagellate *Karenia brevis* (*K. brevis*) is widely distributed in the Gulf of Mexico, and has also been indicated in other locations [1]. Proliferations of *K. brevis* occasionally generates large ’red tide’ blooms. Natural toxins from these blooms cause toxicity to fish, other animals, and human health problems. Brevetoxins (BTXs), actively biosynthesized by *K. brevis,* are the first example of polyether compounds [2]. BTXs consist of more than nine congeners, which are divided into two types (types A and B) on the basis of their polyether backbone structure. The principal toxins in *K. brevis* are the A-types BTX-1, -7, and -10, and B-types BTX-2, -3, -5, -6, -8, and -9 [3]. BTX-1 is the strongest toxin among these toxins, and BTX-1, BTX-2, and BTX-3 are more common than other kinds of brevetoxins in nature. Ingestion of brevetoxin-contaminated shellfish causes neurotoxic shellfish poisoning (NSP). Shellfish contaminated with low concentration of BTXs can cause a nonlethal form of food poisoning, and high concentration of BTXs also affect fish, birds, and marine mammals, causing massive epizootic events [4]. Since these toxins have negative impacts on seafood industries and detrimental effects on human health, it has raised global awareness to develop practical and sensitive detection methods [2,5,6].

In the past years, mouse bioassay as the traditional method often applied to assess toxicity. This means that all the toxins are first measured by subjecting them to animal bioassays [7]. Due to the animal rights concerns, the number and cost of animals required, and some other limitations in the mouse toxicity assay method, other useful techniques for detection of brevetoxins are needed. So far, newer methods for detection of BTXs in biological samples have been developed, such as liquid chromatography coupled with mass spectrometry and tandem mass spectrometry assay (LC-MS/MS) [8,9], functional pharmacology based assay [1], immunoassay [10], and colloidal gold probe-based immune chromatographic assay [11]. LC-MS based assays and other instrumental methods are sensitive to detect marine toxin contamination in seafood samples. However, these methods have some disadvantages, such as complex pre-treatment procedures, and time-consuming, laborious, and expensive costs [12]. Therefore, the ideal method for detection of marine toxins should be simple, rapid, cost-effective, and highly sensitive.

In fact, immunological methods with the advantage of high sensitivity, rapidity, and low-cost become as the most attractive approach for practical purposes. Monoclonal antibody (mAb) with high specificity and affinity is widely used in immunological methods [13,14,15]. The enzyme-linked immune sorbent assay (ELISA) technique has been widely used for diagnostic, residue, and marine toxins detection, which is sensitive, economical, simple, and convenient. Colloidal gold nanoparticles (AuNPs) are visible and can be detected with the naked eye. Therefore, AuNPs conjugate with mAb to develop AuNPs-antibody become one of the most popular methods in immunochromatographic assay. There are some reports about mAb for BTX-2 [16] and BTX-3 [11], which belongs to BTX-B type. To our knowledge, there are still no published articles about the detection of BTX-1 (BTX-A) based on mAb. The purpose of our study was to obtain high-specificity and -affinity mAb against BTX-1 toxin. Then, indirect competitive ELISA (ic-ELISA) and colloidal gold strips based on the mAb were established for the detection of BTX-1 toxin.

## 2. Results and Discussion

### 2.1. Conjugates Preparation and Animal Immunization

**Figure 1:**
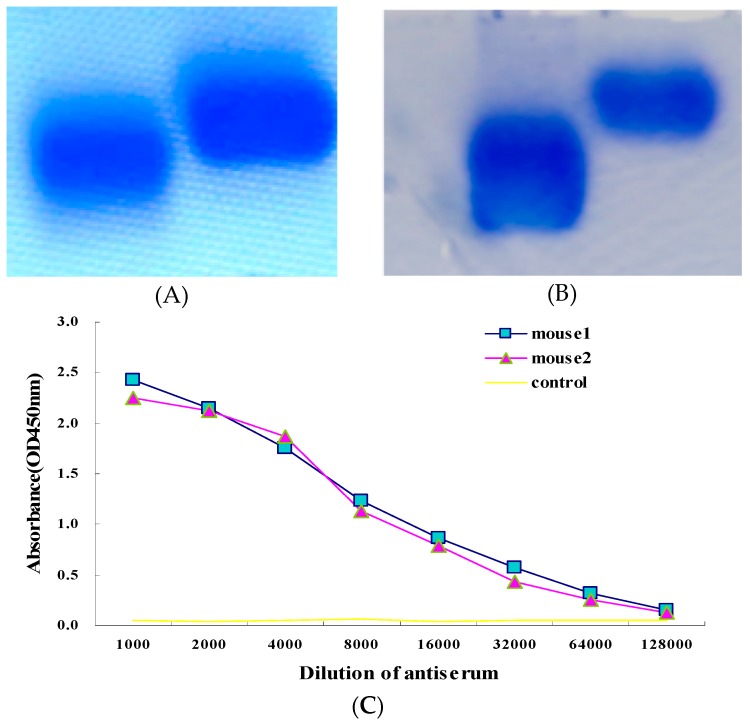
Identification of the conjugates and anti-serum titer. (**A**,**B**) Analysis of conjugates and carrier protein by non-denaturing agarose electrophoris. (**A**) Lane 1: BTX-1-BSA conjugates sample, Lane 2: BSA; (**B**) Lane 1: BTX-1-OVA conjugates sample, Lane 2: OVA. (**C**) Titer of mice anti-serum measured by indirect ELISA. Mouse 1 and 2 were immunized with BTX-1-OVA conjugate, and the control was immunized with only adjuvant and PBS.

BTX-1 is a small molecular weight marine toxin without immunogenicity. In order to obtain a mAb against BTX-1, BTX-1 should be coupled to a carrier protein. Bovine serum albumin (BSA) and ovalbumin (OVA) are frequently used as carrier proteins for conjugate preparation, and BTX-1-BSA and BTX-1-OVA conjugates were prepared by the succinic anhydride method and isobutyl chloroformate method. The agarose gel electrophoresis results of conjugates in Figure 1A,B showed that the migration velocity of BTX-1-BSA and BTX-1-OVA conjugates were faster than that of the carrier protein alone, indicating that BTX-1 was successfully conjugated to the BSA and OVA carrier proteins, respectively. BTX-1-OVA conjugate as an immune antigen was used to immunize health female BALB/c mice that could generate antibody against BTX-1, while BTX-1-BSA was used as a coating antigen for determining the anti-serum titer and in subsequent ELISA experiments.

The titer result showed that the OD value of blood samples from BALB/c mice immunized with BTX-1-OVA was significantly higher than that of the control (Figure 1C).The result further suggested that BTX-1-OVA complete antigen was successful in inducing the antibody against BTX-1. Therefore, the spleen cells of these two mice were selected to perform cell fusion experiments.

### 2.2. Screening and Characterization of Positive Hybridoma Cell Line

**Figure 2:**
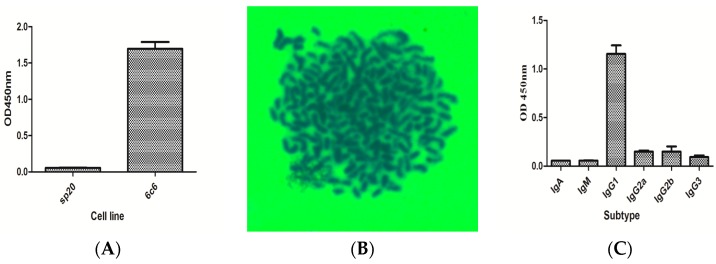
Screening and characterization of positive hybridoma cell against BTX-1. (**A**) The supernatant titer of the positive cell line 6C6 is 1.5, and the supernatant of SP2/0 is selected as a negative control; (**B**) the number of positive hybridoma cell chromosomes is 104; (**C**) the subtype of antibody is IgG1.

In this study, spleen cells from an immunized BALB/c mouse were fused with SP2/0 myeloma cells at the ratio of 10:1 by polyethylene glycol (PEG, 1450). After cell fusion, cells were cultured in HAT medium. After 9 d, the titer of culture supernatant was measured by indirect ELISA. After three times sub-cloning, a positive cell line named 6C6 secreting mAb against BTX-1 was obtained successfully (Figure 2A). In this study, PEG was used for cell fusion. However, the fusion rate of cells and the positive rate of fusion cells were low. There are many factors affecting the cell fusion, such as the growth state of myeloma cells, the concentration of the PEG, the ratio between spleen cells and myeloma cells, and the temperature of the medium. The good-growth state of SP2/0 myeloma cells is one of the most important factors that affects cell fusion. It is very harmful for cells with high concentration of PEG and long incubation time. In this study, 30% PEG was used for cell fusion, there is just 1 min for PEG incubation in the process of cell fusion, and good fusion results were obtained.

Hybridoma cell line 6C6 was chosen for chromosome analysis to further identify whether hybridoma 6C6 was from the fusion of SP2/0 myeloma cell and spleen cell. The average chromosome number of SP2/0 myeloma cell and spleen cell were 62–70 and 38–40, respectively [10]. The chromosome number of the hybridoma 6C6 was counted as 104 (Figure 2B), indicating that cell fusion was successfully carried out. 

The isotype of the positive mAb against BTX-1 was identified by subtype kit (IgM, IgA, IgG1, IgG2a, IgG2b, IgG3), and the result indicated that the subtype of 6C6 positive clone belongs to IgG1 subtype (Figure 2C).

### 2.3. Purification of Anti-BTX-1 mAb and Titer Analysis

**Figure 3:**
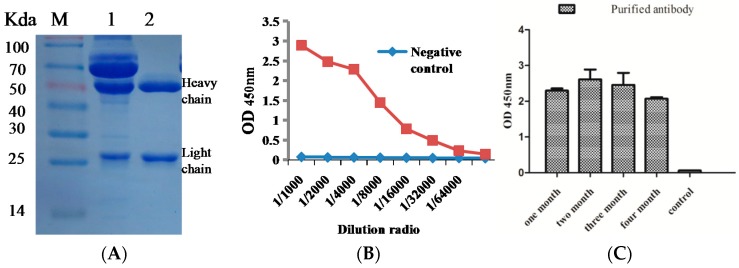
Purification and titer of the anti-BTX-1 mAb. (**A**) SDS-PAGE of the purified anti-BTX-1 mAb. Lane M: maker, Lane 1: ascites, Lane 2: the purified antibody; (**B**) The titer of the purified anti-BTX-1 mAb was identified by indirect ELISA. (**C**) The purified anti-BTX-1 mAb was saved in −20 °C and the titer was identified by indirect ELISA at different times.

Hybridoma cell line 6C6 were cultured and injected into the abdominal cavity of BALB/c mice to prepare more mAb. The anti-BTX-1 mAb was obtained from ascites and purified by caprylic/ammonium sulfate precipitation (CA-AS) method. The purified anti-BTX-1 mAb was analyzed by SDS-PAGE, and the result indicated that the purified antibody has one clear heavy chain at 50 kDa and the light chain at 26 kDa (Figure 3A). The titer of the purified anti-BTX-1 mAb was measured by indirect ELISA, and the result showed that the titer was above 3.2 × 10^4^ (Figure 3B). The result indicated that the antibody was successfully purified with high titer, and could have potential to develop kit for BTX-1 detection. The purified anti-BTX-1 mAb was saved in a refrigerator at −20 °C for a long time, and the titer of the purified anti-BTX-1 mAb was determined by indirect ELISA at the same concentration, and the result indicated that the antibody could retain activity for at least four months (Figure 3C).

### 2.4. Specialty and Affinity of the Anti-BTX-1 mAb

**Figure 4:**
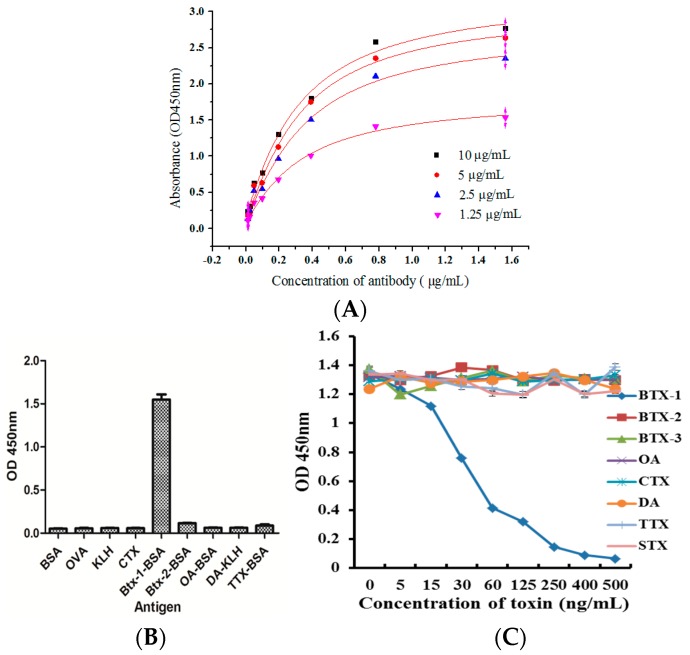
The affinity, specificity, and the cross-reactivity of the anti-BTX-1 mAb were tested by ELISA. (**A**) Affinity was determined by indirect ELISA. Different concentrations of coating antigen was used to determine the affinity constant by indirect ELISA, and the affinity constant is 1.055 × 10^8^ L/mol; (**B**) The specificity analysis of the purified antibody. Different kinds of complete antigen were coated, and the antibody did not react with other antigens; (**C**) cross-reactivity of anti-BTX-1 mAb to other toxins was determined by ic-ELISA. The antibody did not have the cross-reaction with other marine toxins.

The affinity constant of 6C6 mAb against BTX-1 was 1.06 × 10^8^ L/mol (Figure 4A), suggesting that the anti-BTX-1 mAb is highly sensitive to BTX-1. Competitive ELISA was performed to analyze the specificity of the anti-BTX-1 mAb produced by positive clone 6C6, and the ELISA result indicated that 6C6 was highly specific to BTX-1 without any reaction to other complete antigens (Figure 4B). At the same time, anti-BTX-1 mAb showed no cross-reactions between BTX-2, BTX-3, and other marine toxins (Figure 4C) (Table S1). Therefore, it could be further used to develop an ELISA kit for BTX-1 detection.

### 2.5. Standard Curve and Samples Detection by ic-ELISA

**Figure 5:**
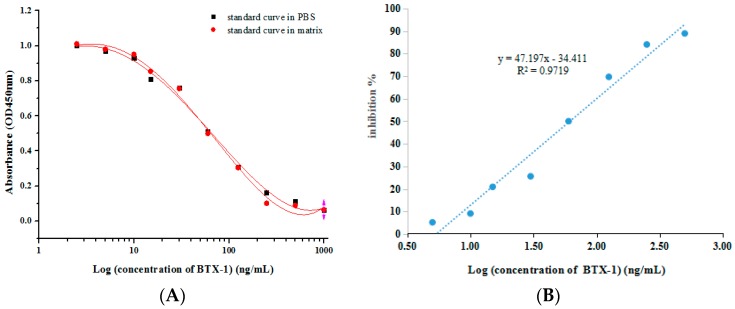
Development of detection method based on ic-ELISA. (**A**) A standard curve was made by competitive inhibition ELISA. The logistic equation was *y* = 0.08779 + (0.098917 − 0.08779)/[1 + (x/58.00982)1.60278], with a correlation coefficient (*R*^2^) of 0.98078. The half inhibitory concentration (IC_50_) of BTX-1 binding to anti-BTX-1 mAb was 60 ng/mL; (**B**) the linear equation is *y* = 47.197*x* − 34.411, with a correlation coefficient (*R*^2^) of 0.0.9719.

The 6C6 mAb was used to establish a standard curve for BTX-1 detection by competitive inhibition ELISA. In the study, BTX-1 was diluted in PBS or matrix, and methanol-water (5:5, *v*/*v*) was used as extraction solution. A standard curve prepared by diluted matrix was compared to a standard curve prepared in PBS, and the two curves showed little difference (Figure 5A), confirming that the matrix was minimized. The relationship between concentration of BTX-1 and inhibition value was analyzed using OriGinpro 8. As shown in Figure 5A, the logistic curve equation was *y* = 0.08779 + (0.098917 − 0.08779)/[1 + (*x*/58.00982)^1.58278^], with a correlation coefficient (*R*^2^) of 0.98078. The half inhibitory concentration (IC_50_) of BTX-1 binding to anti-BTX-1 mAb was 60 ng/mL, and the linear range to detect BTX-1 was 14–263 ng/mL, which defined as the concentration of BTX-1 toward from 20% to 80% inhibition ratio. The linear equation is *y* = 47.197*x* − 34.411, with a correlation coefficient (*R*^2^) of 0.0.9719 (Figure 5B), and the detection limit (LOD) was 14 ng/mL.

Recovery and coefficient of variation (CV) were measured by the ic-ELISA, and shellfish samples without any contamination were spiked with different concentrations of BTX-1. The result showed that the recovery rates were ranged from 84.21 ± 2.15% to 93.11 ± 1.26% with the average of 88 ± 2%, and the variation coefficient is 1.35–2.97% (average 2%) in intra-assay, while the recovery rates were ranged from 85.55 ± 2.05% to 91.55 ± 1.03% with the average of 89 ± 2%, and the variation coefficient is 1.01–4.74% (average 2%) in inter-assay (Table S2). Meanwhile, Shellfish samples (razor, clam mussel, oyster, and scallop) were obtained from a market, and the shellfish sample extracts were diluted 20-fold with phosphate-buffered saline and determined by ic-ELISA. As the results showed in Table S3, there was no BTX-1 in these samples.

### 2.6. Construction and Characterization of Colloidal Gold Strip Test

**Figure 6:**
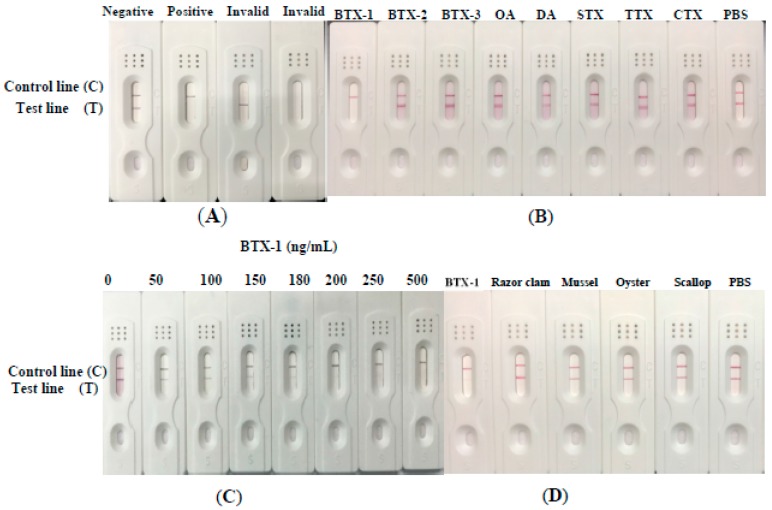
Construction and characterization of colloidal gold strip test; (**A**) the description of strip test results. In the absence of BTX-1 in the sample solution, both two line exits on the control and test zone, indicating negative. Only one control line stands positive for enough toxin binding to the anti-BTX-1-BSA McAb. If no lines, or only the test line was red, it indicated invalid results; (**B**) cross-reactivity of the test strip with other toxins, such as OA, DA, BTX-2, BTX-3, TTX, CTX, STX; (**C**) the detection limit of colloidal gold strip test for BTX-1; (**D**) the real sample solution detection of colloidal gold strip for BTX-1.

The schematic diagram for constructing colloidal gold strip was shown in Figure 6A. There will be two lines in the absence of BTX-1 in the sample solution as a negative control. On the contrary, if the sample solution has enough BTX-1, the test line will disappear and only one control line exists on the control zone. The reason was that BTX-1 will bind with antibody-gold nanoparticle conjugates, so it makes the antibody-gold nanoparticle conjugates fail to bind with the BTX-1-BSA that was coated onto the Millipore 135 NC membrane (Jieyi biotech Co., shanghai, china). Thus, it is shown as a positive control. If there is one test line or no line, it indicates an invalid result. 

Different kinds of toxins, including BTX-1, BTX-2, BTX-3, okadaic acid (OA), domoic acid (DA), saxitoxin (STX), tetrodotoxin (TTX), and conopeptide (CTX), were used to identify the cross-reactivity of the test strip. The result from Figure 6B showed that there were two lines with the existence of other toxins and only one control line with the existence of BTX-1. This result indicated that the colloidal gold strip test based on anti-BTX-1 mAb had high specificity to BTX-1. Different concentrations of BTX-1 solution (0–250 ng/mL) were used to determine the limit of the colloidal gold strip. The results showed that the limit of detection (LOD) of the strip for BTX-1 was 200 ng/mL (Figure 6C), and the test line will disappear totally if the concentration was above this level. Even though the LOD of the colloidal gold strip was higher than that of ELISA, the colloidal gold strip is more convenient, easy to operate, and requires a shorter time than ELISA.

### 2.7. Real Sample Assay with Colloidal Gold Strip

Four kinds of real sample solutions were used to identify whether the collected samples had BTX-1 or not. The result showed that the color density of four test lines of sample solution were the same as the negative control (Figure 6D), indicating that there was no BTX-1 in these four samples. Therefore, this anti-BTX-1 mAb with high affinity and specificity can develop the ELISA kit for detection of BTX-1 in real samples. It also has the potential to develop the colloidal gold rapid diagnostic stripe.

## 3. Conclusions

In summary, a hybridoma cell line 6C6 stably secreting mAb against BTX-1 was obtained. The titer of the antibody was more than 1:32,000 and the affinity of the mAb was 1.06 × 10^8^ L/mol. ELISA and colloidal gold strip assays for BTX-1 were developed based on the mAb against BTX-1. The linear range of ELISA to detect BTX-1 was 14–263 ng/mL with IC_50_ of 60 ng/mL, and the LOD was 14 ng/mL. The average recovery rate of ELISA from the spiked samples is 88 ± 2% in intra-assay and 89 ± 2% in inter-assay. The LOD for colloidal gold strip assay was 200 ng/mL. All these results indicated that the anti-BTX-1 mAb excreted by hybridoma 6C6 could be used to detect BTX-1 in shellfish and other related samples.

## 4. Materials and Methods

### 4.1. Materials

Brevetoxins were purchased from Taiwan Algal Science Inc. (Algal Science Inc., Taiwan, China). Okadaic acid (OA), conopeptide (CTX), polyethylene glycol 1450 (PEG 1450), tetrodotoxin (TTX), HAT medium supplement, HT medium supplement, Mouse monoclonal Antibody Subtyping Kit, tetramethyl benzidine (TMB), Horseradish peroxidase (HRP) conjugated goat anti-mouse IgG, and RPMI 1640 were purchased from Sigma-Aldrich Co. Ltd. (St. Louis, MO, USA). Other reagents were chemical grade from Sinopharm Chemical Regent Co. Ltd. (Beijing, China). Female BALB/c mice were purchased from Wushi Animal Laboratory (Shanghai, China). Myeloma cells were stored in our lab. All animal studies and the procedures used in this study were approved by the Research Ethics Committee, the Fujian Key Laboratory of Pathogenic Fungi Mycotoxins, Fujian Agriculture and Forestry University, Fujian, China (Permit No. PFMFAFU201610). Approval date was 1 November 2016.

### 4.2. Preparation and Analysis of Complete Antigens

BTX-1-OVA and BTX-BSA complete antigens were prepared by two-steps approach (succinic anhydride method and isobutyl chloroformate method) according to the guidebook [16]. Agarose gel electrophoresis (0.8%, non-denaturing) was used to verify whether the conjugation was successful or not [17].

### 4.3. Production of Monoclonal Antibody

BTX-1-OVA was used as an immune antigen. BTX-1-OVA was emulsified with an equal volume of Freund’s complete adjuvant, and then injected into female BALB/c mice (6–8 weeks old) at multiple sites subcutaneously. After eight injections, the titer of the antiserum from the immunized mouse was tested by indirect ELISA [17]. Indirect ELISA steps re described as follows: After coating with BTX-1-BSA antigen, plates were washed with phosphate buffer solution (PBS) and blocked with 5% PBSM (PBS containing 5% defatted milk, 200 µL/well) at 37 °C for 2 h. Then, 100 µL serially-diluted antiserum was added into the plates and incubated for 1 h at 37 °C. Subsequently, plates were washed three times with PBS and PBST (PBS containing 0.5% Tween 20), respectively, and incubated with 100 μL HRP conjugated goat anti-mouse IgG (1:8000 dilution) per well for 1 h at 37 °C. Following, the plates were washed again, and then TMB was added into the well and incubated for 10 min at 37 °C. After that, 2 M H_2_SO4 was added into the wells to stop the reaction, and the absorbance was measured immediately at 450 nm by a microplate reader (Thermo Fisher Scientific, Waltham, MA, USA).

Spleen cells were isolated and collected from the immunized mice with high titer serum, and then fused with SP2/0 myeloma cells at the ratio of 10:1 by polyethylene glycol (PEG, 1450). Cell fusion was carried out according to the guidebook [18]. The hybridoma was subcloned by the limiting dilution method [19].

### 4.4. Analysis of Hybridam Antibody

Chromosome number of the hybridoma cell was analyzed according to the guidebook [20]. Chromosome of hybridoma cell was counted after the treatment of colchicine. Hybridoma cells were treated with 0.4 μg/mL colchicine and harvested by centrifugation, and then resuspended in 10 mL of 0.075 mol/L potassium chloride hypotonic solution. Cells were gathered again by centrifugation and then resuspended with stationary liquid (methanol: acetic acid, *v*/*v* 3:1). The cells were dropped on the glass, and stained with Giemsa stain (St. Louis, MO, USA). Then, the chromosomes can be observed by microscope.

The subtype of the mAb against BTX-1 was tested with a Mouse Monoclonal Antibody Subtyping Kit (IgG1, IgG2b, IgG2a, IgG3, IgA, IgM). Six kinds of antibody subtyping reagents were diluted with PBS, and the solution was dropped into plate for incubation at 37 °C for 1 h following the same procedure as indirect ELISA. 

### 4.5. Purification of mAb and Titre of the Antibody

Mature female BALB/c mice were injected with hybridoma cells (5 × 10^6^ cells) suspended in RPMI 1640, and ascites fluid was collected from abdomen swelled mice through the needle of a 20 mL injector after 8 d injection. The antibody was purified from ascites by caprylic/ammonium sulfate precipitation method [21], and the purified antibody was analyzed by SDS-PAGE [22]. The titer of the purified anti-BTX-1 mAb was measured by indirect ELISA. The detailed information about indirect ELISA was the same as describe above.

### 4.6. Specificity and Affinity of the Purified mAb

The specificity and affinity of this mAb against BTX-1 was determined by indirect ELISA and indirect competitive ELISA, respectively [19]. Firstly, coating antigens BTX-1-BSA, brevetoxin-2 conjugated BSA (BTX-2-BSA), tetrodotoxin-conjugated BSA (TTX-BSA), conopeptide (CTX), okadaic-acid-conjugated BSA (OA-BSA), OVA, BSA, keyhole limpet hemocyanin (KLH), and domoic acid-conjugated KLH (DA-KLH) were diluted to 0.5 μg/mL and added into the plate with 100 μL per well for 1 h at 37 °C. Following operation were the same as indirect ELISA. The cross-reactivity of mAb against BTX-1 was determined by indirect competitive ELISA. Briefly, BTX-1, BTX-2, BTX-3, BTX-7, OA, DA, TTX, CTX, and saxitoxin (STX) (St. Louis, MO, USA) were used as binding competitors, and every competition with different concentrations were mixed with mAb against BTX-1 for 30 min. Then, the above mixture was added into the plate. HRP and TMB were added into the plate, respectively, in the next steps. The absorbance of each well were measured by instrument (Thermo Fisher Scientific, Waltham, MA, USA) after the reaction was stopped by 2 M H_2_SO_4_. Then the cross-reactivity was calculated as: cross-reactivity (CR) = [50% inhibitory concentration (BXT-1)]/[50% inhibitory concentration (competitor)].

The affinity of monoclonal antibody against BTX-1 was determined by indirect ELISA. Different concentrations of complete antigen BTX-1-BSA (10, 5, 2.5, 1.25 μg/mL) were coated into the plate, and the later procedures were the same as the indirect ELISA described above. The absorbance value of each well was measured at 450 nm by a microplate reader after the reaction was stopped by 2 M H_2_SO4. The curve diagram was made and the affinity constant of the antibody was calculated.

### 4.7. Standard Curve and Real Samples Detection by ic-ELISA

Firstly, BTX-1-BSA antigen was added into plates and blocked with PBSM (PBS containing 5% defatted milk), and different concentrations of BTX-1were mixed with the anti-BTX-1 antibody and added into plates. Then HRP and TMB were added into the plate subsequently, and the absorbance of each well was measured at 450 nm after the reaction was stopped by 2 M H_2_SO_4_. The standard curve was made and analyzed using OriGinpro 8 (OriginLab, Northampton, MA, USA). The linear range to detect BTX-1 was defined as the concentration of BTX-1 toward from 20% to 80% inhibition [23]. The matrix effect was minimized by diluting the samples before the ELISA assay, and matrix interference was measured by comparing a standard curve prepared in PBS buffer alone [19].

A recovery study was carried out to determine the efficacy of the standard curve. Different shellfish samples were purchased from local markets, and 10 g of each sample was ground into homogenization. The extraction solution of samples was used in the ELISA assay [17]. The recovery and coefficient of variation values were determined by the spiked samples with different concentrations of BTX-1 with six repeats. Then, the concentration of BTX-1 was detected by the developed ic-ELISA.

### 4.8. Construction and Characterization of Strip Test 

Colloidal gold nanoparticles with a mean particle diameter of 40 nm were used to produce antibody-colloidal gold probes in our study. Then, well-dispersed colloidal gold particles were conjugated with anti-BTX-1 mAb. There are four parts in the strip test, including the sample, conjugate, absorbent pads, and nitrocellulose (NC) membrane with test and control zones [24]. The BTX-1-BSA conjugate was coated onto the Millipore 135 NC membrane as a test line, and HRP-labeled rabbit anti-mouse IgG antibody was coated onto the Millipore 135 NC membrane as a control line. Colloidal gold-antibody conjugates were applied on the treated conjugate pad at a proper spray rate. Finally, four sections of the strip were assembled and stored at room temperature until use.

The competitive immunoassay was performed on the strip test to identify the cross-activity of the colloid gold strip. Different kinds of toxins including BTX-1, BTX-2, BTX-3, okadaic acid (OA), domoic acid (DA), saxitoxin (STX), tetrodotoxin (TTX), and conopeptide (CTX) were used to react with the colloidal gold-BTX-1 mAb conjugate which was pipetted onto the conjugate pad. The detection results could be observed by the naked eye after the mixtures moved forward to the nitrocellulose membrane for incubation for 10 min at room temperature. To evaluate the sensitivity of the strip test, different concentrations of BTX-1 were applied to the sample pad of individual test strips, so that they would flow along the nitrocellulose strip, and then a visible limit of detection (LOD) that resulted in the disappearance of a red band on the test line would be determined [25].

### 4.9. Assay of BTX-1 in Samples with a Colloid Gold Strip

Different kinds of real samples were pretreated as above, and 100 µL of extracted solution was used to react with the colloidal gold-BTX-1 mAb conjugate which was pipetted onto conjugate pad [26]. The detection results could be observed by the naked eye after the mixtures moved forward to the nitrocellulose membrane. After reaction for 5–10 min, the result could be determined whether the related sample contained BTX-1 or not. If both the test and control lines turn red on the NC membrane, the sample is recorded as negative. When the control line but not the test line was colored red, it is considered as positive. In any assay, a red color band should always appear on the control line to make sure the strip test is working properly.

## Supplementary Materials

The following are available online at http://www.mdpi.com/2072-6651/10/2/75/s1, Table S1: The cross-reactivity of anti-BTX-1 mcAb, Table S2: The recovery and coefficient of variation was detected in shellfish samples with spiked toxin, Table S3: BTX-1was detected in some real samples by ic-ELISA.

## References

[1] David L.S., Plakas S.M., Said K.R.E., Jester E.L.E., Dickey R.W., Nicholson R.A. (2003). Nicholson, A rapid assay for the brevetoxin group of sodium channel activators based on fluorescence monitoring of synaptoneurosomal membrane potential. Toxicon.

[2] Abraham A., Plakasa S.M., Wanga Z., Jestera E.L.E., El Saida K.R., Granadea H.R., Henry H.S., Blumb P.C., Pierce R.H., Dickey R.W. (2006). Characterization of polar brevetoxin derivatives isolated from *Karenia brevis* cultures and natural blooms. Toxicon.

[3] Mendoza W.G., Mead R.N., Brand L.E., Shea D. (2008). Determination of brevetoxin in recent marine sediments. Chemosphere.

[4] Naar J., Bourdelais A., Tomas C., Kubanek J., Whitney P.L., Flewelling L., Steidinger K., Lancaster J., Baden D.G. (2002). A Competitive ELISA to Detect Brevetoxins from *Karenia brevis* (Formerly Gymnodinium breve) in Seawater, Shellfish, and Mammalian Body Fluid. Environ. Health Perspect..

[5] Legrand A.M., Litaudon M., Genthon J.N., Bagnis R., Yasumoto T. (1989). Isolation and some properties of ciguatoxin. J. Appl. Phycol..

[6] Lewis R.J., Vernoux J.P., Berreton I.M. (1998). Structure of Caribbean Ciguatoxin Isolated from Caranx latus. J. Am. Chem. Soc..

[7] Wong C.K., Hung P., Kam K.M. (2013). Development of an ICR Mouse Bioassay for Toxicity Evaluation in Neurotoxic Poisoning Toxins-Contaminated Shellfish. Biomed. Environ. Sci..

[8] Nozawa A., Tsuji K., Ishida H. (2003). Ishida, Implication of brevetoxin B1 and PbTx-3 in neurotoxic shellfish poisoning in New Zealand by isolation and quantitative determination with liquid chromatography-tandem mass spectrometry. Toxicon.

[9] Wang Z.H., Steven M.P., Kathleen R.E.S., Edward L.E.J., Granade H.R., Robert W.D. (2004). LC/MS analysis of brevetoxin metabolites in the Eastern oyster (*Crassostrea virginica*). Toxicon.

[10] Kreuzer M.P., Pravda M., O’Sullivan C.K., Guilbault G.G. (2002). Novel electrochemical immunosensors for seafood toxin analysis. Toxicon.

[11] Zhou Y., Pan F.G., Li Y.S., Zhang Y.Y., Zhang J.H., Lu S.Y., Ren H.L., Liu Z.S. (2009). Colloidal gold probe-based immunochromatographic assay for the rapid detection of brevetoxins in fishery product samples. Biosens. Bioelectron..

[12] Liu B.H., Hung C.T., Lu C.C., Chou H.N., Yu F.Y. (2014). Production of Monoclonal Antibody for Okadaic Acid and Its Utilization in an Ultrasensitive Enzyme-Linked Immunosorbent Assay and One-Step Immunochromatographic Strip. J. Agric. Food Chem..

[13] Guan D., Li P.W., Cui Y.H., Zhang Q., Zhang W. (2011). A competitive immunoassay with a surrogate calibrator curve for aflatoxin M1 in milk. Anal. Chim. Acta.

[14] Lei H., He Z., Yuan H., Wu J., Wen L., Li R., Zhang M., Yuan L., Yuan Z. (2012). Generation and characterization of a monoclonal antibody to penicillic acid from *Penicillium cyclopium*. Afr. J. Biotechnol..

[15] Li Y.S., Zhou Y., Lu S.Y., Guo D.J., Ren H.L., Meng X.M., Zhi B.H., Lin C., Wang Z., Li X.B. (2011). Development of a one-step test strip for rapid screening of fumonisins B1, B2 and B3 in maize. Food Control.

[16] Naar J., Branaa P., Bottein-Dechraoui M.Y., Chinain M., Pauillac S. (2001). Polyclonal and monoclonal antibodies to PbTx-2-type brevetoxins using minute amount of hapten-protein conjugates obtained in areversed micellar medium. Toxicon.

[17] Jin N., Ling S.M., Yang C., Wang S.H. (2014). Preparation and identification of monoclonal antibody against Citreoviridin and development of detection by Ic-ELISA. Toxicon.

[18] Zhang A.H., Ma Y.N., Feng L.L., Wang Y., He C.C., Wang X.H., Zhang H.B. (2011). Development of a sensitive competitive indirect ELISA method for determination of ochratoxin A levels in cereals originating from Nanjing. China. Food Control.

[19] Ling S.M., Pang J., Yu J.J., Wang R.Z., Liu L.C., Ma Y.L., Zhang Y.M., Jin N., Wang S.H. (2014). Preparation and identification of monoclonal antibody against fumonisin B1 and development of detection by Ic-ELISA. Toxicon.

[20] Lin K.Q., Wen Y., Chu J.Y. (2010). The method and significance of chromosome analysis in the study of hybridoma. Chin. Med. Biotechnol..

[21] Bai L., Qian J.F., Wang J. (2000). Extration IgG antibodies from ascites and serum of mice by ammonium sulphate method. J. Da Li Med. Coll..

[22] Wang R.Z., Fang S., Xiang S.S., Ling S.M., Yuan J., Wang S.S. (2014). Generation and Characterization of a scFv Antibody Against T3SS Needle of *Vibrio parahaemolyticus*. Indian J. Microbiol..

[23] Kido K., Edakuni K., Morinaga O., Tanaka H., Shoyama Y. (2008). An enzyme-linked immunosorbent assay for aconitine-type alkaloids using an anti-aconitine monoclonal antibody. Anal. Chim. Acta.

[24] Wang Z.Z., Zhi D.J., Zhao Y., Zhang H.L., Wang X., Ru Y., Li H.Y. (2014). Lateral flow test strip based on colloidal selenium immunoassay for rapid detection of melamine in milk, milk powder, and animal feed. Int. J. Nanomed..

[25] Ling S.M., Chen Q.A., Zhang Y.M., Wang R.Z., Jin N., Pang J., Wang S.H. (2015). Development of ELISA and colloidal gold immunoassay for tetrodotoxin detetcion based on monoclonal antibody. Biosens. Bioelectron..

[26] Ling S.M., Wang R.Z., Gu X.S., Wen C., Chen L.L., Chen Z.B., Chen Q.A., Xiao S.W., Yang Y.L., Zhuang Z.H. (2015). Rapid detection of fumonisin B1 using a colloidal gold immunoassay strip test in corn samples. Toxicon.

