# Supplementary material for: Preparation of Monoclonal Antibody for Brevetoxin 1 and Development of Ic-ELISA and Colloidal Gold Strip to Detect Brevetoxin 1
Source: Toxins (Basel). 2018 Feb 8;10(2):75. doi: 10.3390/toxins10020075 (PMC5848176; doi:10.3390/toxins10020075)
Supplement: Supplementary file 1 [file toxins-10-00075-s001.pdf]

Supplementary Materials: Preparation of Monoclonal Antibody for Brevetoxin 1 and Development of Ic-ELISA and Colloidal Gold Strip to Detect Brevetoxin 1

Sumei Ling, Shiwei Xiao, Chengjie Xie, Rongzhi Wang, Linmao Zeng, Ke Wang, Danping Zhang, Xiulan Li and Shihua Wang

Table S1. The cross-reactivity of anti-BTX-1 mcAb.

BTX-1	BTX-2	BTX-3	OA	CTX	DA	TTX	STX
100%	<0.1%	<0.1%	<0.1%	<0.1%	<0.1%	<0.1%	<0.1%

Table S2. The recovery and coefficient of variation was detected in shellfish samples with spiked toxin.

Spiked level (ng/mL)	Inter-assay* (n = 3)			Intra-assay# (n = 3)		
	Measured (ng/mL)	Recovery (%)	CV (%)	Measured (ng/mL)	Recovery (%)	CV (%)
150	139.67 ± 1.89	93.11 ± 1.26	1.35	137.33 ± 1.55	91.55 ± 1.03	1.13
100	91.87 ± 1.59	91.88 ± 1.59	1.73	90.16 ± 0.91	90.16 ± 0.91	1.01
50	44.03 ± 1.31	88.06 ± 2.62	2.97	45.36 ± 2.15	90.72 ± 4.30	4.74
20	16.84 ± 0.43	84..21 ± 2.15	2.68	17.11 ± 0.41	85.55 ± 2.05	2.4
14	11.88 ± 0.26	84.86 ± 1.86	2.11	12.06 ± 0.39	86.14 ± 2.79	3.23
Average		88 ± 2	2		89 ± 2	2

* The first day; # The fourth day.

Table S3. BTX-1was detected in some real samples by ic-ELISA.

Sample	OD 450 nm Value	Results
PBS	1.324	--
razor	1.358	--
clam mussel	1.321	--
oyster	1.353	--
scallop	1.336	--

-- mean no toxin
